# Detection of *Dirofilaria immitis* in golden jackals (*Canis aureus* L.) but not in red foxes (*Vulpes vulpes* L.) and European badgers (*Meles meles* L.) in Croatia

**DOI:** 10.1186/s13071-024-06576-z

**Published:** 2024-11-27

**Authors:** Šimun Naletilić, Ema Gagović, Željko Mihaljević, Adam Polkinghorne, Ana Beck, Relja Beck

**Affiliations:** 1 Department of Pathology, Croatian Veterinary Institute, Savska cesta 143, 10000 Zagreb, Croatia; 2 Department for Bacteriology and Parasitology, Croatian Veterinary Institute, Savska cesta 143, 10000 Zagreb, Croatia; 3 Microbiology, New South Wales Health Pathology, Nepean Hospital, Penrith, NSW 2751 Australia; 4 Nepean Clinical School, Faculty of Medicine and Health, University of Sydney, 62 Derby St, Kingswood, NSW 2747 Australia; 5 O- zna, Ribnjak 8, 10000 Zagreb, Croatia

**Keywords:** Canine heartworm disease, Dirofilariosis, Wild carnivores, Surveillance

## Abstract

**Background:**

Dirofilariosis is a parasitic mosquito-borne disease caused by members of the genus *Dirofilaria*, which includes *Dirofilaria immitis* and *Dirofilaria* repens. Surveillance studies in Europe have revealed that *D. immitis* can also be detected in a range of wild carnivores, raising questions over the impact of infections on wild carnivore animal health but also whether these populations may act as a reservoir for infection of other species, including domestic dogs and humans.

**Methods:**

In the current study, we conducted surveillance for the presence of *D. immitis* in several wild carnivore species, including golden jackals (*Canis aureus*; *n* = 77), red foxes (*Vulpes vulpes*; *n* = 326), and European badgers (*Meles meles;* *n* = 28), collected during an annual rabies surveillance and control program from across continental and coastal regions of Croatia.

**Results:**

Macroscopic examination of the right chambers of the heart during a post-mortem examination resulted in the detection of filarial parasites in 6.5% (5/77) golden jackal carcasses. Morphological identification, confirmed by molecular screening, classified all parasites as *D. immitis*. No *D. immitis* were detected in the red foxes or European badgers examined. All infected golden jackals were adults aged from 2 to 7 years with a parasite load ranging from 2 to 7 nematodes per carcass. One animal was infected with a sexually mature pair, while a second harbored pre-mature parasites; the remaining positive jackals were infected with female parasites only. Notably, histological examination of cardiac and lung tissue revealed proliferative endarteritis in the jackal with the highest parasite burden.

**Conclusions:**

Further studies are required to establish whether golden jackals, as well as other wild carnivore hosts, may serve as competent definitive hosts of *D. immitis* and act as reservoirs for infection of other species including domestic dogs and humans. Histological changes in the cardiac tissue of at least one positive jackal were suggestive of infection with pathological consequences for the host.

**Graphical Abstract:**

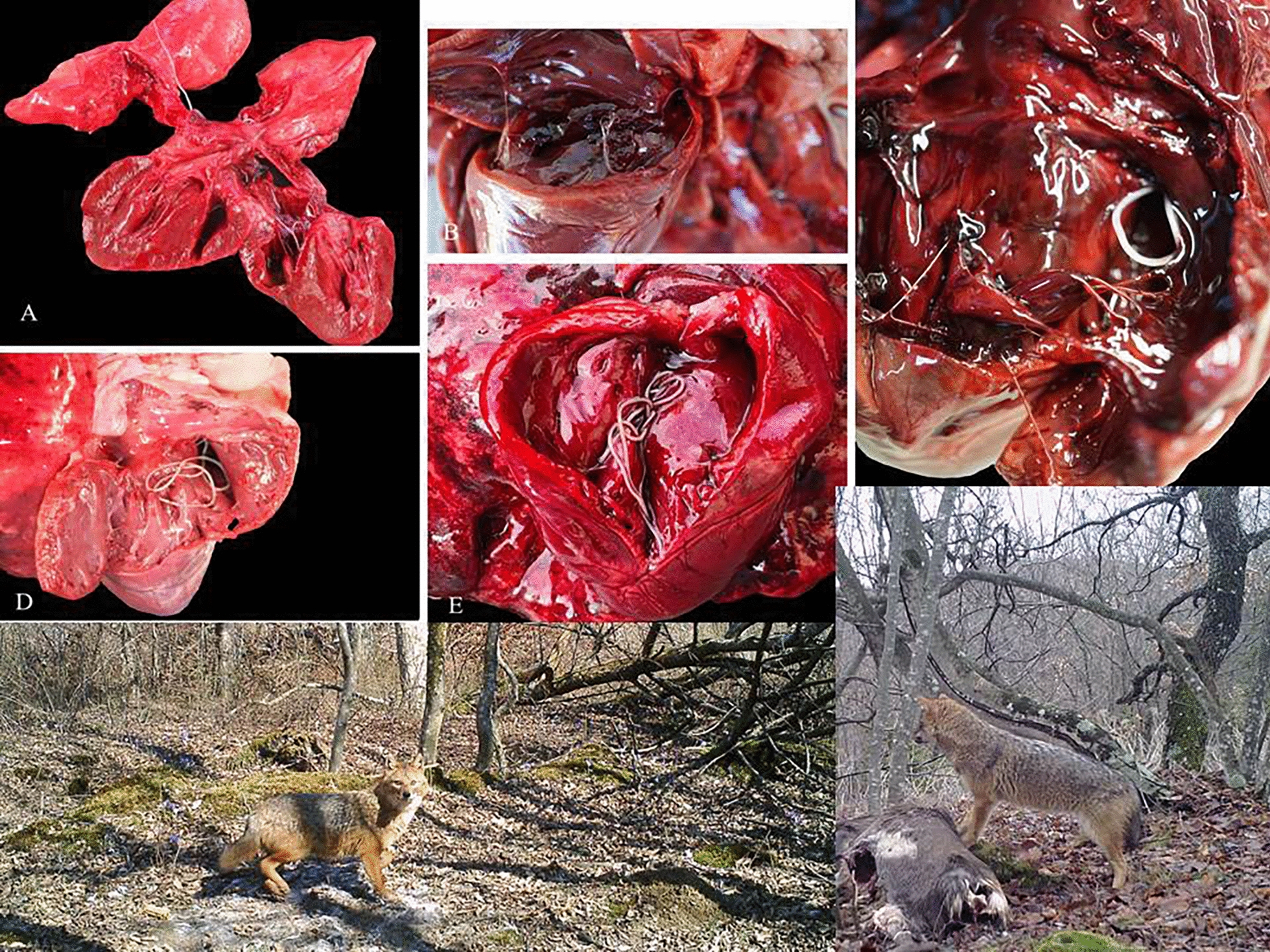

**Supplementary Information:**

The online version contains supplementary material available at 10.1186/s13071-024-06576-z.

## Background

Dirofilariosis is a vector-borne disease caused by filaroid nematodes, mainly *Dirofilaria immitis* and *Dirofilaria repens*. Infections are most commonly described in dogs, where infections are generally asymptomatic. Less commonly, infection can give rise to subcutaneous nodules (e.g., *D. repens*; subcutaneous dirofilariosis) or result in fatal cardiopulmonary disease (e.g., *D. immitis*; heartworm disease). Infections are also observed in humans. *Dirofilaria repens* is usually the cause of subcutaneous and ocular dirofilariosis in humans and is characterized by a migration that results in inflammatory reactions or nodule formation, including the development of microfilaremia [1–3]. Parasites can occasionally be found in a variety of organs and tissues, including the genitalia, breasts, lungs, abdominal cavity, and muscles. *Dirofilaria immitis* is mostly responsible for pulmonary dirofilariosis, which is characterized by the production of nodules, but it can also be present in subcutaneous tissues, the eyes, and internal organs [1, 2]. Infection between hosts is mediated by invasive and autochthonous mosquito species, including females of the genera *Culex*, *Aedes*, *Ochlerotatus*, *Anopheles*, and *Mansonia* [4–6], which serve as intermediate hosts and vectors. *Dirofilaria immitis* is present in many countries across the globe [7] and is most commonly detected in temperate and tropical to subtropical climatic regions [8]. Southern Europe is considered an endemic area for *D. immitis*; however, climate change and migration of vector populations is suspected to contribute to the geographical spread of dirofilariosis through the rest of the continent [9–11].

Besides dogs, more than 30 mammalian species worldwide have been reported as hosts for *D. immitis*. In Europe, *D. immitis* has been detected in wolves (*Canis lupus*) [12, 13], badgers (*Meles meles*) [14], European wildcats (*Felis silvestris*) [13, 15], European otters (*Lutra lutra*) [15, 16], raccoon dogs (*Nyctereutes procyonoides*) [14], and ferrets (*Mustela putorius furo*) [17]. *Dirofilaria immitis* infections have also been reported in red foxes (*Vulpes vulpes*) [13, 15, 18–20] and golden jackals (*Canis aureus*) [8, 13, 15, 18–21], two species whose populations are expanding across Europe. In the case of golden jackals, in particular, this expansion includes Croatia and the suburban areas of Croatian towns and cities [22].

The expanding distribution of wild carnivores such as red foxes and golden jackals has raised important questions about the *D. immitis* infection status of these hosts in Croatia and other countries in this region. To date, surveillance studies in countries neighboring Croatia suggest that *D. immitis* is prevalent in red foxes and golden jackals. In Hungary, the prevalence of *D. immitis* in red foxes and golden jackals has been reported as 3.7% and 7.4%, respectively [19]. In Serbia, *D. immitis* infections were detected in the heart and pulmonary arteries of 1.6% of red foxes and 7.3% of golden jackals [13], with higher prevalence rates of 8.5% (red foxes) and 6.3% (golden jackals) reported following molecular screening of blood specimens [23]. Romanian studies identified adults of *D. immitis* in red foxes (6.6%) and golden jackals (18.5–19.1%), with a subsequent molecular study of spleen samples [14, 21] from these hosts revealing a generally lower prevalence than reported earlier (red foxes—0.3%; golden jackals—7.6%) [15]. In Bulgaria, Panayotova-Pencheva et al. [20] reported *D. immitis* prevalence of 37.5%, 25.2%, and 33.3% in golden jackals, red foxes, and domestic dogs, respectively. Finally, in a single study from Bosnia and Herzegovina, necropsy and molecular screening of blood specimens failed to detect any *D. immitis* from red foxes [24].

Previous Croatian studies on the seroprevalence of *D. immitis* in dogs have suggested a very low prevalence of 0.4–0.6% [25–27]. No studies to date have been conducted in wild carnivores. In the current study, we attempted to address this knowledge gap by conducting a post-mortem examination and molecular screening of several wild carnivore species retrieved from across Croatia.

## Methods

From September 2021 to March 2023, 431 wild carnivore carcasses collected as part of an annual rabies surveillance and control program were submitted for post-mortem examination to the Laboratory for Pathology of the Croatian Veterinary Institute, Zagreb, Croatia. These submissions included 326 red foxes (*V. vulpes*), 77 golden jackals (*C. aureus*), and 28 badgers (*M. meles*). All animals were collected after legal hunting or had died following motor vehicle trauma. They originated from 11 counties across Croatia including Zagreb County, Krapina-Zagorje, Varaždin, Međimurje, Karlovac, Lika-Senj, Sisak-Moslavina, Bjelovar-Bilogora, Požega-Slavonia, Brod-Posavina, and Dubrovnik-Neretva (see Additional file 1). The carcasses were examined immediately upon arrival, and animal location, sex, and estimated age were recorded. The age of animals was determined through the analysis of canine tooth cementum lines as part of a surveillance program [28, 29].

The cardiopulmonary systems of all animals were macroscopically examined for the presence of *D. immitis* adults using a systematic approach from the left to the right side of the heart. Each heart was opened using a small horizontal incision to the left atrium, after which a larger vertical incision was made along the core of the left ventricle in the direction of the apex of the heart. The two cuts were then joined with scissors. A similar procedure was repeated on the right side of the heart, after which the pulmonary artery was opened with scissors. All detected filarial nematodes were collected and identified based on morphological keys and morphometric data available in the literature [30, 31]. The number of *D. immitis*, gender, and developmental stage were noted. Furthermore, a portion of the uterus from each female parasite examined was placed on a microscope slide with lactophenol and covered with a cover slip for microfilariae visualization. Blood samples were collected from the hearts of infected golden jackals and analyzed for *Dirofilaria* antigen presence using the SNAP 4Dx (IDEXX) test.

A section of the right ventricle, pulmonary artery, and caudal lung lobe were fixed in 10% buffered formalin for 24 h. A standard protocol for routine histology was followed, including dehydration, embedding in paraffin blocks, and cutting of serial 4-µm-thick slices. The sections were stained with hematoxylin and eosin (H&E) and prepared for examination under a microscope (Zeiss Axio Imager.A2). Microscope images were captured with a Digicyte BigEye microscope camera.

A single *D. immitis* nematode from each golden jackal carcass underwent molecular confirmation of morphological identification. DNA was extracted from *D. immitis* using the DNA blood and tissue kit (Qiagen, Hilden, Germany) in the QIAcube automatic extraction system (Qiagen, Hilden, Germany). Conventional polymerase chain reaction (PCR) assays were used for confirmation of the identity of *D. immitis* following sequencing of a 667-base-pair (bp) region of the cytochrome *c* oxidase I (*COI*) gene, as previously performed by Casiraghi et al. [12]. PCRs were performed in total volumes of 20 µl using 1 µl of extracted DNA. The amplified products were analyzed by capillary electrophoresis (QIAxcel System^®^, Qiagen) with size markers in the range of 100–2500 bp. Samples were purified with ExoSAP-IT^®^ (USB Corp., Cleveland, OH, USA) and sequenced in both directions by Macrogen, Inc. (Netherlands). Sequences were assembled using SeqMan Pro software, edited with the EditSeq tool of Lasergene software (DNASTAR, Madison WI, USA), and compared with available sequences using the Basic Local Alignment Search Tool (BLAST).

## Results

**Fig. 1:**
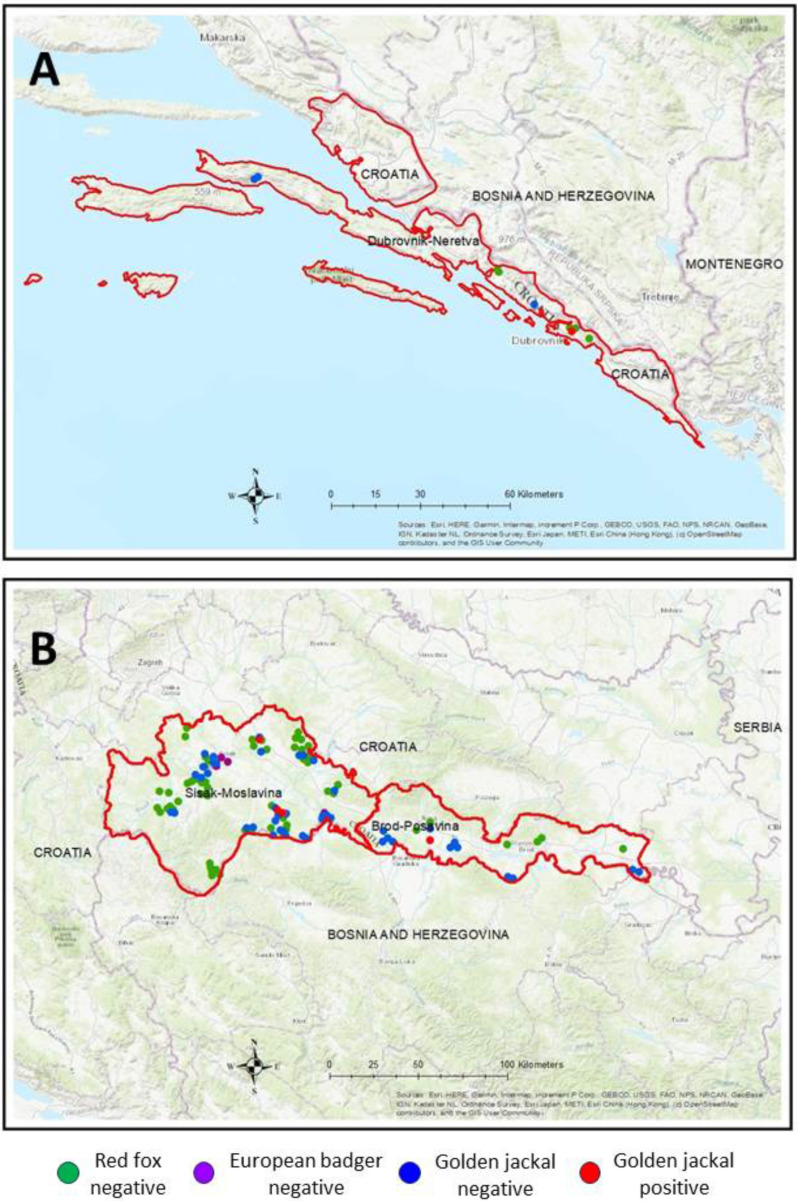
A map of the counties with infected golden jackals, showing the locations of all the infected animals

Following post-mortem examination, 5/431 (1.2%; CI 0.038–2.686) wild carnivore carcasses collected from across Croatia were found to be positive for *D. immitis* (Table 1). All five positive cases (5/77, 6.5%; CI 2.14–14.51) were detected in golden jackals; no *D. immitis* were detected in any badger (0/28; 0.0%) or red fox carcasses (0/326; 0.0%) examined. Infected golden jackals originated from three counties, Sisak-Moslavina (9.1%; CI 1.91–24.33), Brod-Posavina (7.7%; CI 0.19–36.03), and Dubrovnik-Neretva (25.0%; CI 0.63–80.59) (Fig. 1 and Table 1). Chronologically, the first infected golden jackal was confirmed in June 2022 from Sisak-Moslavina county, and the last sample was collected from an animal originating from Brod-Posavina in February 2023 (Table 2).

**Table 1:** Dirofilarial positivity in wild carnivores sampled from across Croatia between September 2021 and March 2023

County	Red fox *Vulpes vulpes* (%)	European badger *Meles meles* (%)	Golden jackal *Canis aureus* (%)
Zagreb County	0/61 (0.0)	0/7 (0.0)	0/11 (0.0)
Krapina-Zagorje	0/41 (0.0)	0/7 (0.0)	0/1 (0.0)
Varaždin	0/32 (0.0)	0/1 (0.0)	0/0 (0.0)
Međimurje	0/5 (0.0)	0/0 (0.0)	0/0 (0.0)
Karlovac	0/50 (0.0)	0/0 (0.0)	0/12 (0.0)
Lika-Senj	0/35 (0.0)	0/5 (0.0)	0/1 (0.0)
Sisak-Moslavina	0/52 (0.0)	0/4 (0.0)	3/33 (9.09, CI 1.91–24.33)
Bjelovar-Bilogora	0/18 (0.0)	0/1 (0.0)	0/1 (0.0)
Požega-Slavonia	0/22 (0.0)	0/3 (0.0)	0/1 (0.0)
Brod-Posavina	0/5 (0.0)	0/0 (0.0)	1/13 (7.69 CI 0.19–36.03)
Dubrovnik-Neretva	0/5 (0.0)	0/0 (0.0)	1/4 (25.0 CI 0.63–80.59)

**Table 2:** Characteristics of the *D. immitis*-positive golden jackals from this study

Animal ID	Date collected	County	Gender/age	Number of parasites	Gender of parasites	Antigen detection	Gross lesions	Histological lesions
1	June 2022	Sisak-Moslavina	Male, 2–3 years	7	Six females, one male	Positive	No	Yes
2	November 2022	Sisak-Moslavina	Female, 2–3 years	4	Pre-adult	Negative	No	No
3	November 2022	Sisak-Moslavina	Female, 3–4 years	2	Females	Positive	No	No
4	December 2022	Dubrovnik-Neretva	Female, 6–7 years	4	Females	Positive	No	No
5	February 2023	Brod-Posavina	Male, 2–3 years	2	Females	Positive	No	No

All infected golden jackals were adults ranging in age from 2 to 7 years, with a sex ratio of 3:2 in favor of females. An average of 3.8 parasites were detected per carcass, ranging from two to seven nematodes across the five positive golden jackal carcasses. Only one animal was infected with a sexually mature pair (Table 2), one was harboring premature parasites, and three were only infected with female parasites. Apart from one animal which was positive for premature parasites, the remaining four parasite-positive golden jackals were positive for circulating antigen following SNAP 4Dx testing of blood extracts (Table 2).

**Fig. 2:**
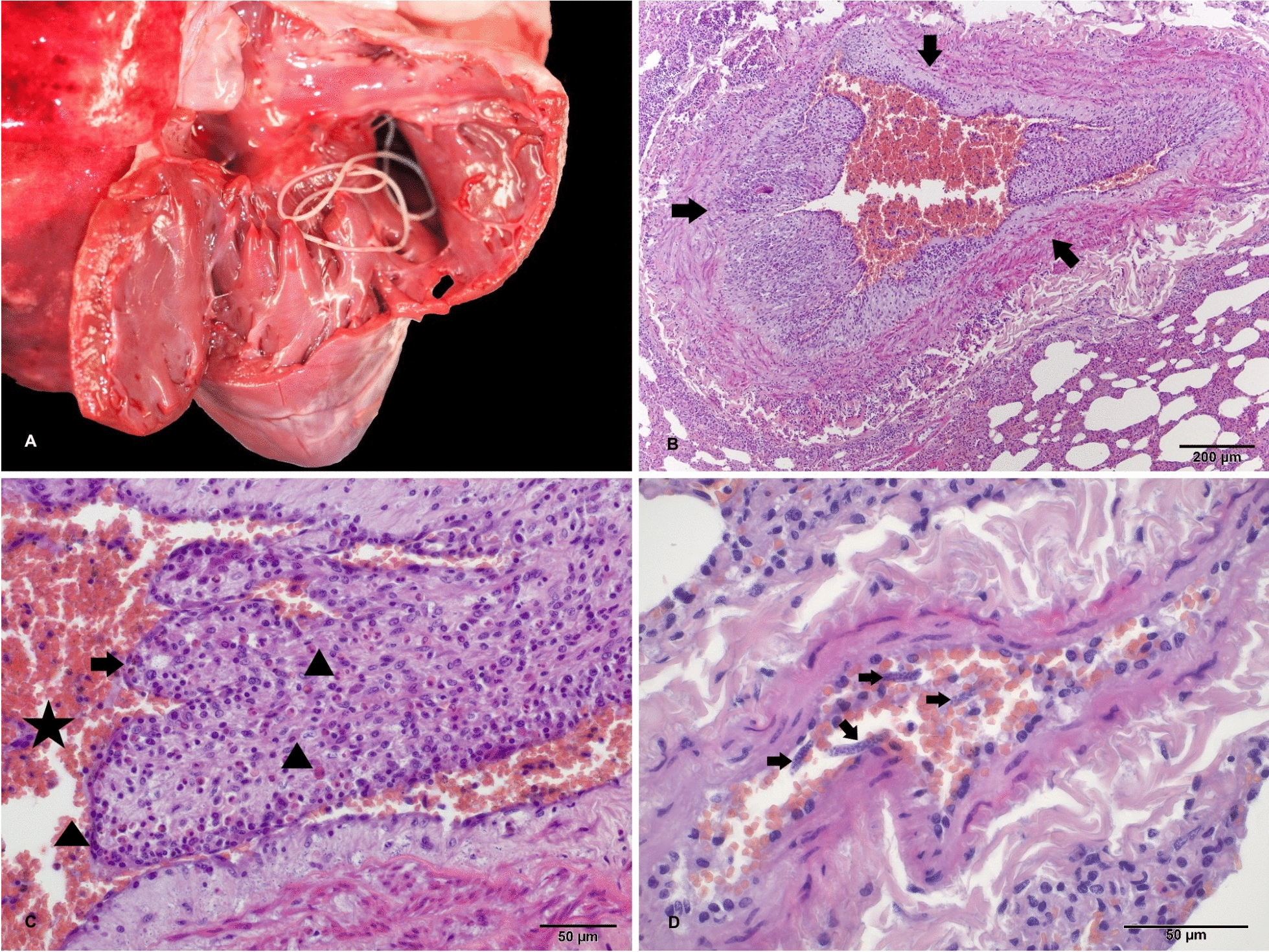
Golden jackal, lungs and right side of the heart opened, exposing a white adult nematode measuring 20 cm × 4 mm (*D. immitis*) extending from the ventricle into the pulmonary artery (**A**). Pulmonary artery, transverse section with irregularly narrowed lumen (arrows); adjacent alveolar tissue with emphysema and hemorrhage due to gunshot trauma (**B**). Lumen of artery (star) with villous endarteritis (arrows) characterized by extensive myofibrosis and eosinophilic, partially histiocytic, and neutrophilic inflammation (arrowheads) (**C**). Medium-sized pulmonary artery harboring four longitudinal and tangential sections of microfilariae admixed with erythrocytes (arrows) (**D**). Hematoxylin and eosin (H&E) stain

No visible lesions were detected on gross examination of the hearts in infected animals with a special focus on evidence of hypertrophy or alterations associated with right heart failure. Microscopic lesions in the form of multifocal proliferative endarteritis were detected only in the branches of the pulmonary lobe arteries (Fig. 2) in Animal 1, a male estimated to be 2–3 years old and with the highest number of parasites (Table 2). The lumina of the pulmonary arteries was obscured by prominent villar folds composed of loosely arranged collagen and/or myofibers lined by 1–2 layers of mildly hypertrophic endothelium. The intima was infiltrated with moderately dense accumulations of eosinophils admixed with a few histiocytes, neutrophils, and lymphocytes. The tunica muscularis was discreetly thickened due to smooth muscle hypertrophy. Longitudinal, tangential, and rarely transverse sections of microfilariae were observed within the lumen of medium-sized arteries. No alterations were detected in the capillary endothelium. Microscopic lesions were not observed in any of the other golden jackals infected with *D. immitis* (Table 2).

All nematodes were identified based on the morphology as *D. immitis*. This morphological identification was further confirmed by sequencing of the 667-bp portion of the *COI* gene from each animal. All sequences were identical to each other and to *D. immitis* sequences deposited from Western Europe (Spain—LC107816; Italy—FN391553) and elsewhere (Australia—NC005305). A representative sequence was deposited in the GenBank database under accession number PQ045537.

## Discussion

The golden jackal is a mesocarnivore that has rapidly expanded its range across Europe, colonizing new habitats in Central and South-Central Europe [32]. In Croatia, the golden jackal’s range includes regions along the southwestern Adriatic coastline, with distinct populations found inland to the east and separated by the Dinaric Alps. Despite the increasing number of studies on the presence and distribution of *D. immitis* in wild carnivores, data are still missing from certain areas, including Croatia. The present study, which aimed to identify *D. immitis* in wild carnivore populations in Croatia, provides the first evidence for the presence of this parasite in golden jackals in different regions of this country.

In our study, the detection of *D. immitis* in hearts collected post-mortem revealed an overall prevalence of 1.2% among all examined species. In contrast to studies from neighboring countries, including Romania, Bulgaria, Serbia, and Hungary [13–15, 19, 23], *D. immitis* was only detected in golden jackals, with no parasites detected in the hearts of red foxes or badgers. In Croatian golden jackals, the overall prevalence was 6.5%. This prevalence was similar to that reported in several studies using the same screening strategy. In Serbia, the *D. immitis* prevalence was 7.3% [13] while a Hungarian study detected this parasite in 7.4% of golden jackals [19]. This positivity was nevertheless noticeably lower than that in Romanian (18.5–19.1%) [14, 21] and Bulgarian studies (37.5%) [20]. The failure to detect *D. immitis* in badger carcasses is perhaps unsurprising given that microfilaremia was only recently documented in this species for the first time in a Romanian study, with only one individual positive among 115 specimens (0.9%) [14]. More unexpected was the lack of evidence for *D. immitis* in Croatian red foxes. However, it should be noted that in other studies of wild carnivores in Eastern Europe, the prevalence of *D. immitis* in red foxes appears to be consistently lower than rates reported in golden jackals [13–15, 20]. The confirmation of the presence of *D. immitis* in golden jackals in Croatia adds impetus to answering important questions about the role of wild carnivores in the maintenance and spread of this helminth parasite. This is still a matter of debate, and it is yet unclear whether wildlife may act as a reservoir [13, 33–35], a sentinel [14], or an accidental host for this parasite. Furthermore, while it has now been reported in a variety of wild carnivores, which species, if any, play important roles in the sylvatic cycle of *D. immitis* [14] is still an open question.

To further understand the *D. immitis* infection status in golden jackals from Croatia and their reservoir capacity, we also determined the number, sex, developmental stage, and presence of microfilariae in female parasites. In the present study, the average number of worms per animal was 3.8 (ranging from 2 to 7), which is comparable to the previously reported worm burden of 3 and 2.9 in golden jackals [14, 21]. The evidence supporting the claim that wild animals generally have a lower parasite burden than domestic canines is demonstrated by the average prevalence of 1.5 in red foxes [14] and 5 (with a range of 3–7) in wolves [12]. Conversely, dogs from the USA exhibited a significantly higher average parasite count, reaching 14 (with a range of 1–85) [36] and 23 (with a range of 1–131) [37]. In most of the previous studies on golden jackals, patency of the parasite was not in the scope of the studies [15] and, when evaluated, microfilariae in female parasites were not present [19]. The finding of only female parasites in three animals, juvenile parasites in one, and females with microfilaria in only one animal provides some evidence to suggest that jackals do not currently play a significant role as a reservoir.

It was interesting to note that the golden jackal with the highest parasite burden (Animal #1; Table 2) was also the only animal with histological changes observed in cardiac tissue. To the best of our knowledge, this is the first report of histological changes caused by *D. immitis* in golden jackals. Dogs with heartworm disease predominantly exhibit changes in the pulmonary vasculature, manifested as myointimal proliferations with villous projection into the arterial lumen [38, 39]. Infiltration of inflammatory cells is present in clinical infection, but subsides later in the course of the disease [40]. This presentation can change depending on the breed, the number of parasites, and the length of the infection. The observed endarteritis in this golden jackal is suggestive of infection with pathological consequences for the host, with histological changes that could potentially have progressed to the death of this animal. The fact that these changes were only found in the animal with the highest parasite burden suggests that, like in dogs, parasite burden is one factor that may influence the outcome of a *D. immitis* infection in golden jackals.

## Conclusions

This study contributes to a growing body of evidence suggesting that *D. immitis* can infect wild carnivores. While these infections appear to be relatively rare, helminthic infection may have consequences for the health of individual animals. Nevertheless, the potential for these hosts to form a part of the life cycle of *D. immitis* and serve as a potential reservoir for infection of other species remains unclear. Further longer-term studies, in which the prevalence of parasites, their number, gender, and microfilaremia will be determined, are required to fully understand the role of wild canids as reservoirs and their role in the spread of *D. immitis*.

## Supplementary Information

Additional file 1.

## Data Availability

No datasets were generated or analyzed during the current study.

## References

[1] Simón F, Siles-Lucas M, Morchón R, González-Miguel J, Mellado I, Carretón E, et al. Human and animal dirofilariasis: the emergence of a zoonotic mosaic. Clin Microbiol Rev. 2012;25:507–44. 22763636 10.1128/CMR.00012-12 PMC3416488

[2] Simón F, Diosdado A, Siles-Lucas M, Kartashev V, González-Miguel J. Human dirofilariosis in the 21st century: a scoping review of clinical of reported in the literature. Transbound Emerg Dis. 2022;69:2424–39. 34197050 10.1111/tbed.14210

[3] Pupić-Bakrač A, Pupić-Bakrač J, Beck A, Jurković D, Polkinghorne A, Beck R. Dirofilaria repens microfilaremia in humans: case description and literature review. One Health. 2021;13:100306. 34466651 10.1016/j.onehlt.2021.100306 PMC8385151

[4] Alsarraf M, Levytska V, Mierzejewska EJ, Poliukhovych V, Rodo A, Alsarraf M, et al. Emerging risk of *Dirofilaria* spp. infection in Northeastern Europe: high prevalence of *Dirofilaria repens* in sled dog kennels from the Baltic countries. Sci Rep. 2021;11:1068. 33441797 10.1038/s41598-020-80208-1 PMC7806926

[5] Latrofa MS, Montarsi F, Ciocchetta S, Annoscia G, Dantas-Torres F, Ravagnan S, et al. Molecular xenomonitoring of *Dirofilaria immitis* and *Dirofilaria repens* in mosquitoes from north-eastern Italy by real-time PCR coupled with melting curve analysis. Parasit Vectors. 2012;5:76. 22520170 10.1186/1756-3305-5-76 PMC3438040

[6] Silaghi C, Beck R, Capelli G, Montarsi F, Mathis A. Development of *Dirofilaria immitis* and *Dirofilaria repens* in *Aedes japonicus* and *Aedes geniculatus*. Parasit Vectors. 2017;10:94. 28219407 10.1186/s13071-017-2015-x PMC5319011

[7] Noack S, Harrington J, Carithers DS, Kaminsky R, Selzer PM. Heartworm disease—overview, intervention, and industry perspective. Int J Parasitol Drugs Drug Resist. 2021;16:65–89. 34030109 10.1016/j.ijpddr.2021.03.004 PMC8163879

[8] Sharifdini M, Karimi M, Ashrafi K, Soleimani M, Mirjalali H. Prevalence and molecular characterization of *Dirofilaria immitis* in road killed canids of northern Iran. BMC Vet Res. 2022;18:161. 35501899 10.1186/s12917-022-03270-z PMC9063217

[9] Genchi C, Rinaldi L, Mortarino M, Genchi M, Cringoli G. Climate and *Dirofilaria* infection in Europe. Vet Parasitol. 2009;163:286–92. 19398159 10.1016/j.vetpar.2009.03.026

[10] Genchi C, Mortarino M, Rinaldi L, Cringoli G, Traldi G, Genchi M. Changing climate and changing vector-borne disease distribution: the example of *Dirofilaria* in Europe. Vet Parasitol. 2011;176:295–9. 21300439 10.1016/j.vetpar.2011.01.012

[11] Genchi C, Kramer LH. The prevalence of *Dirofilaria**immitis* and *D*. *repens* in the Old World. Vet Parasitol. 2020;280:108995. 32155518 10.1016/j.vetpar.2019.108995

[12] Moroni B, Rossi L, Meneguz PG, Orusa R, Zoppi S, Robetto S, et al. *Dirofilaria immitis* in wolves recolonizing northern Italy: are wolves competent hosts? Parasit Vectors. 2020;13:482. 32962753 10.1186/s13071-020-04353-2 PMC7507288

[13] Penezić A, Selaković S, Pavlović I, Ćirović D. First findings and prevalence of adult heartworms (*Dirofilaria immitis*) in wild carnivores from Serbia. Parasitol Res. 2014;113:3281–5. 24951168 10.1007/s00436-014-3991-9

[14] Ionică AM, Deak G, Boncea R, Gherman CM, Mihalca AD. The European badger as a new host for *Dirofilaria immitis* and an update on the distribution of the heartworm in wild carnivores from Romania. Pathogens. 2022;11:420. 35456095 10.3390/pathogens11040420 PMC9032528

[15] Ionică AM, Matei IA, D’Amico G, Ababii J, Daskalaki AA, Sándor AD, et al. Filarioid infections in wild carnivores: a multispecies survey in Romania. Parasites Vectors. 2017;10:332. 28705255 10.1186/s13071-017-2269-3 PMC5508779

[16] Penezić A, Moriano R, Spasić M, Ćirović D. First report of a naturally patent infection with *Dirofilaria immitis* in an otter (*Lutra lutra*). Parasitol Res. 2018;117:929–31. 29374324 10.1007/s00436-018-5769-y

[17] Molnár V, Pazár P, Rigó D, Máthé D, Fok E, Glávits R, et al. Autochthonous *Dirofilaria immitis* infection in a ferret with aberrant larval migration in Europe. J Small Anim Pract. 2010;51:393–6. 20626785 10.1111/j.1748-5827.2010.00950.x

[18] Panayotova-Pencheva M, Šnábel V, Dakova V, Čabanová V, Cavallero S, Trifonova A, et al. *Dirofilaria**immitis* in Bulgaria: the first genetic baseline data and an overview of the current status. Helminthologia. 2020;57:211–8. 32855608 10.2478/helm-2020-0026 PMC7425230

[19] Tolnai Z, Széll Z, Sproch Á, Szeredi L, Sréter T. *Dirofilaria immitis*: an emerging parasite in dogs, red foxes and golden jackals in Hungary. Vet Parasitol. 2014;203:339–42. 24810374 10.1016/j.vetpar.2014.04.004

[20] Panayotova-Pencheva M, Mirchev R, Trifonova A. *Dirofilaria immitis* infection in carnivores from Bulgaria: 2012–2013 update. BJVM. 2016;19:153–62.

[21] Ionică AM, Matei IA, D’Amico G, Daskalaki AA, Juránková J, Ionescu DT, et al. Role of golden jackals (*Canis** aureus*) as natural reservoirs of *Dirofilaria** spp.* in Romania. Parasit Vectors. 2016;9:240. 27121617 10.1186/s13071-016-1524-3 PMC4848770

[22] Sindičić M, Bujanić M, Štimac I, Martinković F, Tuškan N, Špehar M, et al. First identification of *Echinococcus multilocularis* in golden jackals in Croatia. Acta Parasitol. 2018;63:654–6. 29975650 10.1515/ap-2018-0076

[23] Potkonjak A, Rojas A, Gutiérrez R, Nachum-Biala Y, Kleinerman G, Savić S, et al. Molecular survey of *Dirofilaria* species in stray dogs, red foxes and golden jackals from Vojvodina, Serbia. Comp Immunol Microbiol Infect Dis. 2020;68:101409. 31881413 10.1016/j.cimid.2019.101409

[24] Hodžić A, Alić A, Klebić I, Kadrić M, Brianti E, Duscher GG. Red fox (*Vulpes vulpes*) as a potential reservoir host of cardiorespiratory parasites in Bosnia and Herzegovina. Vet Parasitol. 2016;223:63–70. 27198779 10.1016/j.vetpar.2016.04.016

[25] Mrljak V, Kuleš J, Mihaljević Ž, Torti M, Gotić J, Crnogaj M, et al. Prevalence and geographic distribution of vector-borne pathogens in apparently healthy dogs in Croatia. Vector Borne Zoonotic Dis. 2017;17:398–408. 28448211 10.1089/vbz.2016.1990

[26] Jurković D, Beck A, Huber D, Mihaljević Ž, Polkinghorne A, Martinković F, et al. Seroprevalence of vector-borne pathogens in dogs from Croatia. Parasitol Res. 2019;118:347–52. 30377795 10.1007/s00436-018-6129-7

[27] Lovrić L, Vavžik V, Živičnjak T. Subclinical dirofilariosis in dogs in Croatia–results of retrospective research based on archived blood samples. Vet Arh. 2022;92:323–30.

[28] Grue H, Jensen B. Annular structures in canine tooth cementum in red foxes (*Vulpesvulpes* L.) of known age. Dan Rev Game Biol. 1973;8:1–12.

[29] Goddard HN, Reynolds JC. Age determination in the red fox (*Vulpesvulpes* L.) from tooth cementum lines. Gibier Faune Sauvage. 1993;10:173–87.

[30] Manfredi MT, Di Cerbo A, Genchi M. Biology of filarial worms parasitizing dogs and cats. In: Genchi C, Rinaldi L, Cringoli G, editors. *Dirofilaria immitis* and *D. repens* in dog and cat and human infections. Naples: Mappe parassitologiche, Università degli Studi di Napoli Federico II; 2007. p. 39–47.

[31] Lok JB, Harpaz T, Knight DH. Abnormal patterns of embryogenesis in *Dirofilaria **immitis* treated with ivermectin. J Helminthol. 1988;62:175–80. 3192909 10.1017/s0022149x00011482

[32] Stronen AV, Konec M, Boljte B, Bošković I, Gačić D, Galov A, et al. Population genetic structure in a rapidly expanding mesocarnivore: golden jackals in the Dinaric-Pannonian region. Glob Ecol Conserv. 2021;28:e01707.

[33] Gherman CM, Mihalca AD. A synoptic overview of golden jackal parasites reveals high diversity of species. Parasit Vectors. 2017;10:419. 28915831 10.1186/s13071-017-2329-8 PMC5603039

[34] Gavrilović P, Marinković D, Todorović I, Gavrilović A. First report of pneumonia caused by *Angiostrongylus vasorum* in a golden jackal. Acta Parasitol. 2017;62:880–4. 29035862 10.1515/ap-2017-0107

[35] Selanec I, Lauš B, Sindičić M. Golden jackal (*Canis aureus*) distribution in Croatia. Paris: VI European Congress of mammalogy; 2011. p. 60.

[36] Kaiser L, Williams JF. *Dirofilaria immitis*: worm burden and pulmonary artery proliferation in dogs from Michigan (United States). Vet Parasitol. 2004;124:125–9. 15350667 10.1016/j.vetpar.2004.06.015

[37] Courtney CH, Zeng QY. The structure of heartworm populations in dogs and cats in Florida. Charleston: Proceedings of the heartworm symposium; 1989. p. 1–6.

[38] Kawabata A, Nakagaki K, Yoshida M, Shirota K. Histopathological comparison of pulmonary artery lesions between raccoon dogs (*Nyctereutes procyonoides*) and domestic dogs experimentally infected with *Dirofilaria immitis*. J Vet Med Sci. 2008;70:301–3. 18388433 10.1292/jvms.70.301

[39] Atwell RB, Sutton RH, Moodie EW. Pulmonary changes associated with dead filariae (*Dirofilaria immitis*) and concurrent antigenic exposure in dogs. J Comp Pathol. 1988;98:349–61. 3392249 10.1016/0021-9975(88)90043-6

[40] Robinson WF, Robinson NA. Cardiovascular system. In: Maxie MG, editor. Jubb, Kennedy, and Palmer’s pathology of domestic animals. 6th ed. St. Louis: Elsevier; 2016. p. 83–5.

