# Supplementary figures and images for: Detection of Dirofilaria immitis in golden jackals (Canis aureus L.) but not in red foxes (Vulpes vulpes L.) and European badgers (Meles meles L.) in Croatia
Source: Parasit Vectors. 2024 Nov 27;17:490. doi: 10.1186/s13071-024-06576-z (PMC11600906; doi:10.1186/s13071-024-06576-z)

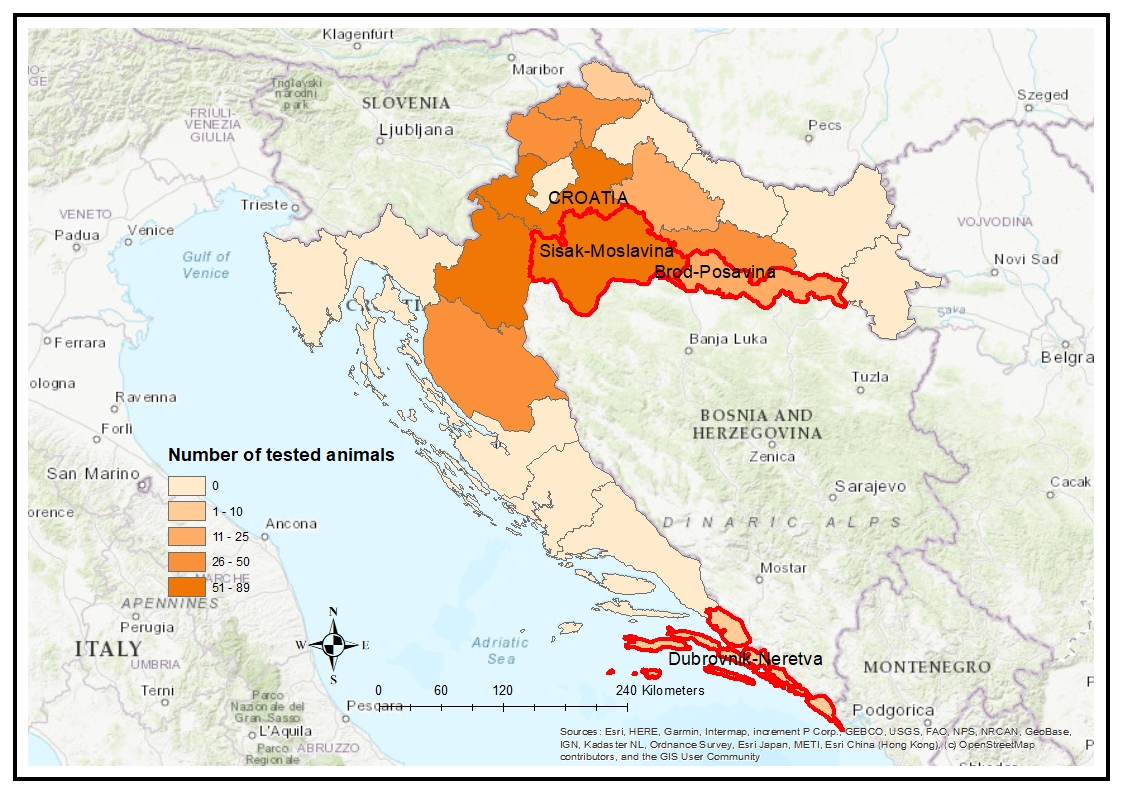

Supplement: Supplementary file 1 — Additional file 1. [file 13071_2024_6576_MOESM1_ESM.jpg]
